# Gastrointestinal perforation caused by displaced contraceptive device: a case report

**DOI:** 10.11604/pamj.2024.49.13.44432

**Published:** 2024-09-11

**Authors:** Hiba Ben Hassine, Mohamed Ali Chaouch, Maissa Jallali, Sadek Ben Jabra, Mabrouk Abdelalli, Ibtissem Korbi, Faouzi Noomen

**Affiliations:** 1 Department of Visceral Surgery, Fattouma Bourguiba Hospital, Monastir University, Monastir, Tunisia,; 2 Department of Radiology, Fattouma Bourguiba Hospital, Monastir University, Monastir, Tunisia

**Keywords:** Contraceptive device, migration, penetration, gastrointestinal perforation, case report

## Abstract

The intrauterine device (IUD) is a widely utilized contraceptive method. In cases of uterine rupture, an IUD can migrate to the intra-abdominal or pelvic cavity, leading to various complications such as gastrointestinal perforation. The aim of this study was to report a case of a 29-year-old woman presented with acute left lumber pain. A computerized tomography scan revealed a left para tubal collection measuring 5x6cm, with the IUD located outside the uterine cavity and penetrating the rectum. Surgical intervention was performed, resulting in the complete retrieval of the device and closure of the perforation. The case report highlights the potential for serious complications associated with IUD use and the need for healthcare providers to be vigilant and to promptly recognize and manage them through a multidisciplinary approach.

## Introduction

The intrauterine device (IUD) is one of the widely used contraception, with a rising rate of adoption year [1]. Although it is an effective method, uterine perforation can occur during or after the procedure of IUD insertion due to the migration of the IUD to the pelvic or intra-abdominal cavity, resulting in various complications such as gastrointestinal perforation [2]. Therefore, the aim of this study was to report a case of gastrointestinal perforation caused by displaced contraceptive device to underscore the role of a multidisciplinary team in avoiding fatal outcomes associated with IUD migration.

## Patient and observation

**Patient information:** a 29-year-old woman presented with acute left lumbar pain.

**Clinical findings:** physical examination revealed tenderness in the left lumbar region.

**Timeline of the current episode:** the patient had no history of previous abdominal or urologic surgery. After undergoing IUD insertion, she received multiple courses of antibiotics for pelvic inflammatory disease.

**Figure 1:**
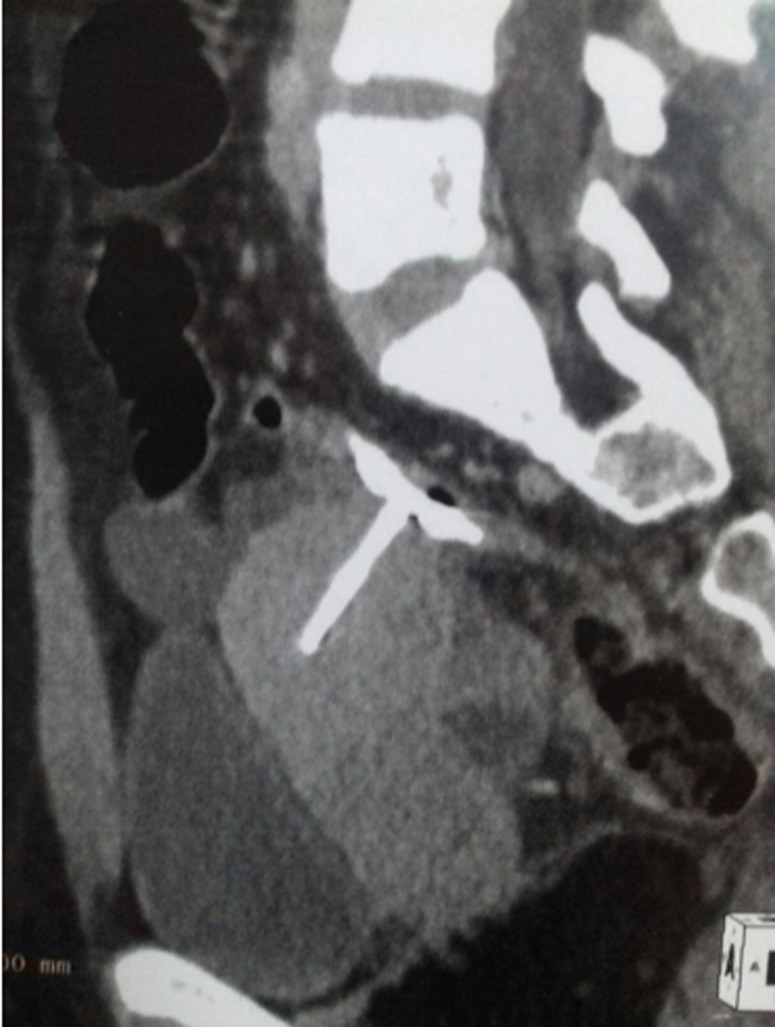
a computerized tomography scan showing the intra uterine device having an ectopic position, which both arms are embedded in the wall of the rectum with a close 5x6cm collection

**Diagnostic assessment:** laboratory investigations indicated a biological inflammatory response, with a white blood cell count of 15,000 and CRP of 125. Pelvic and abdominal computerized tomography scan revealed a 5x6cm left paratubal collection, fat infiltration, and the IUD located outside the uterine cavity with possible penetration into the rectum (Figure 1).

**Diagnosis:** gastrointestinal perforation caused by displaced contraceptive device.

**Figure 2:**
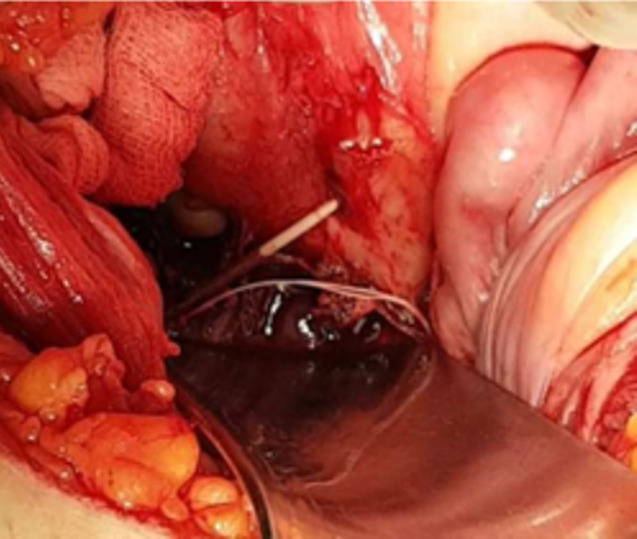
intraoperative images showing a device embedded in the wall of the rectum

**Therapeutic interventions:** pfannenstiel incision, intraoperative exploration revealed a par tubal collection with the IUD incarcerated at the rectum (Figure 2). After abscess evacuation and salpingectomy, the device was completely retrieved, and perforation closure was performed with abdominal cavity drainage.

**Follow-up and outcome of interventions:** the patient's postoperative course was uncomplicated at the 6-month follow-up, she remained asymptomatic. She opted for oral contraception.

**Patient perspective:** the patient expressed optimism for a full recovery.

**Informed consent:** the patient provided written consent for the publication of her clinical details and any identifying images.

## Discussion

The intrauterine device (IUD) is a widely used contraceptive method, it is the third most commonly used according to the World Health Organization (WHO) [1]. Uterine perforation can occur during the procedure or as a delayed event, with an estimated frequency of 1.2 per 1000 insertions [2,3]. Factors influencing the risk of perforation include the type of IUD, insertion technique, operator experience, follow-up diligence, myometrial defects, and the interval between delivery and insertion [4]. In this case, the IUD was inserted five months post-delivery. To mitigate complications, it is recommended that trained clinicians perform IUD placements following good patient selection [5]. Intrauterine device migration can remain asymptomatic in 85% of cases. Ectopic IUDs may manifest late through the disappearance of the string marker or during unintended pregnancies in 30% of cases [6] or with abdominal pain, bleeding, and infertility. Complications of IUD migration include acute intestinal obstruction, fistula, intra-abdominal abscess, or peritonitis. Rectal perforation can be seen in 15-20% of cases [4,7].

Regular gynecological examinations are crucial for monitoring IUD position and detecting potential complications. Follow-up visits at 6-12 weeks post-insertion and then every two years are recommended. In cases of intraperitoneal migration where ultrasound may not provide clear visualization, abdominal pelvic CT imaging is recommended to confirm the ectopic location of the IUD and identify associated complications [8]. The World Health Organization and the International Planned Parenthood Federation advocate for prompt IUD removal upon diagnosis to prevent adhesion formation, chronic pelvic pain, bowel obstruction, or further migration into adjacent organs. Laparoscopy is the preferred surgical approach for ectopic IUD removal due to its minimally invasive nature with success rates ranging from 44% to 100%. However, in cases of severe complications such as sepsis or intestinal perforation requiring repair, conversion to laparotomy may be necessary [6]. In this case, a pfannenstiel laparotomy was performed.

## Conclusion

Intrauterine devices are recognized as safe and effective forms of reversible contraception. Migration of the device can occur, and perforation may be mistakenly diagnosed. Achieving an accurate diagnosis often necessitates additional morphological examination. It is imperative for clinicians to remain vigilant regarding asymptomatic patients and ensure periodic follow-up to monitor device position and detect any potential complications. In cases where an IUD has migrated intra-abdominally, laparoscopic removal or conservative management may be the best course of action.
